# New Insights into Cholesterol Functions: A Friend or an Enemy?

**DOI:** 10.3390/nu11071645

**Published:** 2019-07-18

**Authors:** Antonis Zampelas, Emmanuella Magriplis

**Affiliations:** Department of Food Science and Human Nutrition, Agricultural University of Athens, Iera odos 75, 11855 Athens, Greece

Cholesterol is a sterol synthesized by animal cells and is also a component of the diet, being present in food of animal origin. Its main function is to maintain the integrity and fluidity of cell membranes and to serve as a precursor for the synthesis of substances that are vital for the organism including steroid hormones, bile acids, and vitamin D. Although a high dietary cholesterol intake was considered as a risk factor for cardiovascular diseases (CVD), recent evidence suggests that it does not increase significantly low-density lipoprotein cholesterol (LDL-C) levels in the circulation. Results, however, remain controversial, potentially due to the correlation with saturated fat intake. In view of these recent findings and the fact that cholesterol plays a vital role in major functions in the body, the special issue of *Nutrients* on “Cholesterol and Health” focused on the functions of cholesterol and the effects of dietary cholesterol in various metabolic processes.

Cholesterol and bile acid synthesis have a diurnal rhythm in the body, but the role of the circadian system in cholesterol homeostasis is still being investigated. A better understanding of this system is necessary for the development of targeted interventions to improve metabolic health before considering the effects of dietary cholesterol or the body’s cholesterol as a risk factor for specific diseases, with cardiovascular being the main. A study in this special issue of *Nutrients* systematically reviewed the literature on the diurnal rhythms of cholesterol synthesis and absorption markers, and of bile acid synthesis markers [1]. In addition, it looked at the diurnal rhythms of the cholesterol synthesis markers lathosterol and desmosterol, and the cholesterol absorption markers cholestanol, campesterol, and sitosterol in serum samples from the Bispebjerg Study. The study came to the conclusion that although cholesterol and bile acid synthesis have a diurnal rhythm, no evidence was found for a diurnal rhythm of cholesterol absorption.

Risk factors of CVD are affected by nutrients, foods, dietary patterns, and energy balance. Four very interesting studies in this issue tried to address these aspects [2,3,4,5]. Firstly, data from the Hellenic National Nutrition and Health Survey (HNNHS) indicated and confirmed other studies’ findings that eggs, which are a main source of dietary cholesterol, do not increase the risk of dyslipidemia. The study concluded that eggs could be consumed as part of a healthy diet that is high in fiber and low in saturated fat, without excessive energy intake, by all individuals [2]. Interestingly, the results of the HNNHS actually indicated that the risk of dyslipidemia could even decrease with moderate egg consumption of less than five per week, possibly due to a balanced diet in total.

The second study [3] was carried out during Ramadan, a very important religious period in the Muslim world. It is known that fasting during this period involves large changes in daily eating patterns, which strongly impacts the daily biorhythm and challenges the regular function of the digestive tract. This study assessed the effects of a high fiber cereal at dawn (Sohor) during the month of Ramadan on satiety, bowel habits, body composition, blood glycemia, and blood lipidemia and it was found that this consumption had a positive effect on satiety, and it improved bowel functions and blood lipid levels.

The third paper is a narrative review on selected functions of high density lipoprotein (HDL) particles and ways that various dietary patterns may affect cardiovascular health biomarkers, with a focus on HDL functionality [4]. HDL is involved in the reverse cholesterol transport and high HDL cholesterol levels are considered anti-atherogenic. The review in the present issue suggests that the Mediterranean diet has strong positive contribution to higher HDL cholesterol efflux capacity and paraoxonase 1 activity. Paraxonase 1 is an enzyme which hydrolyzes oxidized lipids in LDL and prevents peroxidation of LDL particles. HDL cholesterol efflux capacity and paraoxinase 1 are also associated with several food groups, such as virgin olive oil, lycopene-rich diet, nuts, and eggs. Some of these foods also play a central part in the Diet Approach to Stop Hypertension (DASH) and other healthful plant-based patterns, but the review suggested that there is limited data on HDL functionality when on these dietary patterns.

Finally, the fourth paper [5] addresses weight loss and its effects on blood lipid profile. It is known that, if weight loss is induced by chronic energy deficit, it could improve blood lipid profile. However, the effects of an acute negative energy balance and the comparative efficacy of diet and exercise are still not well established. In this paper, therefore, the effects of progressive, acute energy deficits (20% or 40% of daily energy requirements) induced by a single day of calorie restriction or aerobic exercise were investigated in healthy subjects. The results of this very interesting study showed that an acute negative energy balance induced by calorie restriction and aerobic exercise could reduce triglyceride levels in a dose-dependent manner by decreasing circulating large and medium very low density lipoprotein (VLDL) particles, which consequently could contribute the decrease of the risk of CVD.

To change someone’s nutritional behavior, primarily nutritional knowledge has to be assessed in order to set methods for improvement. A cross-sectional survey recruiting students in the United Arab Emirates investigated knowledge, attitudes, and practices related to dietary salt intake and a 24-h dietary recall among a subsample of the study population, to assess the dietary intake of total fat, cholesterol, saturated fat, trans fat, and sodium [6]. The results from this study showed that students with low salt-related knowledge scores were correlated with higher prevalence of overweight/obesity as well as hypertension. The results also revealed a high percentage of students exceeding the recommended intake of total fat (48% of sample), saturated fat (90%), trans fat (64%), and sodium (89%), and all students did not meet potassium intake recommendations. The fact that foods high in saturated fat are also good sources of cholesterol indicates the combination may increase risk of CVD, especially in relation to this population’s high salt and low potassium intake. Public health intervention programs to decrease red meat and its products, as well as dietary sodium, are of paramount importance in this region. 

Apart from CVD, chronic kidney disease (CKD) is a major worldwide public health problem that can eventually lead to end-stage renal disease (ESRDA) if it is not controlled. The “Ketodiet” refers to a variety of amino acid ketoanalogues (nitrogen-free analogues of essential amino acids) and low-protein diets (LPDs; 0.6 g/kg per day) or very-low-protein diets (VLPDs; 0.3–0.4 g/kg per day), which allow a reduced intake of nitrogen while avoiding the deleterious consequences of inadequate dietary protein intake and malnourishment. In this issue, a meta-analysis of 12 intervention studies [7] showed that Restricted Protein diets supplemented with ketoanalogues could slow down the progression of CKD in patients with estimated glomerular filtration rate (eGFR) >18 mL/min/1.73 m^2^ without causing malnutrition, and reverse CKD-mineral and bone disorders (MBD) in patients with eGFR < 18 mL/min/1.73 m^2^. As expected, this specific supplementation with ketoanalogues did not affect cholesterol levels. 

Although, for the reduction of chronic disease risk blood cholesterol levels should be low, there are organs in the human body where cholesterol is of major importance, such as the eye. In particular, plasma membranes of the human lens fiber cell are overloaded with cholesterol that saturates the phospholipid bilayer of these membranes and leads to the formation of pure cholesterol bilayer domains. A very interesting review in this issue focused on the beneficial and harmful cholesterol actions in eye lens focusing especially on high cholesterol, which has a different function in the eye lens compared to other tissues and organs [8]. It was emphasized that the major difference between cholesterol action in the lens versus in other tissues and organs is that the eye lens is avascular, and therefore it is not exposed to blood and its related components, including cholesterol transported in LDL and HDL. Additionally, differentiation of lens fiber cells involves formation of an organelle-free zone comprising of cells devoid of these. Since the organelle-free zone in the lens consists of only the plasma membranes and cytosol, the high cholesterol content in the lens seems to be important and beneficial.

Another important aspect that was presented in this cholesterol issue was the association between CVD and bone metabolism [9]. A positive correlation between CVD and osteoporosis risk has been suggested, which implies a close relationship between hyperlipidemia and/or hypercholesterolemia and bone metabolism. Cholesterol and its metabolites influence bone homeostasis through modulating the differentiation and activation of osteoblasts and osteoclasts. In addition, the hematopoietic cells and bone marrow adipocytes take up the majority of space in bone cavity and the effects of cholesterol on hematopoietic stem cells, including proliferation, migration, and differentiation, are also well-known and relate to atherosclerotic lesions. However, correlation between circulating cholesterol and bone marrow adipocytes remains elusive. In the last review article in this special issue, the latest progress on the effects of cholesterol on the regulation of bone metabolism and bone marrow microenvironment, including the hematopoiesis and marrow adiposity, were explored. Some mechanisms were proposed but it was underlined that the functions of bone marrow adipocyte tissue are still widely unknown. It was also noted that future research exploring the physiological and pathological functions of bone marrow adipocyte tissue could provide greater insight on the importance of bone marrow niche homeostasis in local hematopoiesis and osteogenesis, which could potentially act beneficially on therapeutic strategies for atherothrombosis and osteoporosis.

Finally, heated oils may also affect risk of various diseases. It is widely known that heating oils and fats for a considerable length of time results in chemical reactions, leading to the aggravation of free radical processes, which ultimately contributes to the development of atherosclerosis. An animal study in this issue investigated the effects of feeding heated oils with or without dietary cholesterol on the development of atherosclerosis in rabbits, since these animals are considered very good models for atherosclerosis research [10]. The results of this study suggested that heated palm oil could protect against the development of atherosclerosis compared to heated polyunsaturated oils in a rabbit model. This can potentially be explained by the effect that heat has on the chemical structure of polyunsaturated fatty acids due to the presence of double bonds, compared to the absence of double bonds in chemical structure of palm oil and consequently its insusceptibility to oxidation.

This issue covered a great amount of many different aspects of the role of cholesterol in the body. It is understandable that each investigator focused on their specific field of study; therefore, in terms of CVD, the potential negative effect on blood lipids are investigated, compared to researchers investigating eye lens cells, where the beneficial effects of cholesterol are underlined. It is certain that cholesterol is required in the body for many functions and that most evidence suggests that dietary cholesterol alone, in the presence of a healthy diet, does not negatively affect blood lipids. Overall dietary intake and cooking methods, however, can affect this relationship and may potentially be the reason of the controversial finding that are seen to date. This special issue can help future cholesterol investigators plan their study, incorporating parameters provided here, in their plan and analysis. 

## References

[1] Schroor M., Sennels H., Fahrenkrug J., Jørgensen H., Plat J., Mensink R. (2019). Diurnal Variation of Markers for Cholesterol Synthesis, Cholesterol Absorption, and Bile Acid Synthesis: A Systematic Review and the Bispebjerg Study of Diurnal Variations. Nutrients.

[2] Magriplis E., Mitsopoulou A., Karageorgou D., Bakogianni I., Dimakopoulos I., Micha R., Michas G., Chourdakis M., Chrousos G., Roma E. (2019). Frequency and Quantity of Egg Intake Is Not Associated with Dyslipidemia: The Hellenic National Nutrition and Health Survey (HNNHS). Nutrients.

[3] Jarrar A., Beasley J., Ohuma E., Cheikh Ismail L., Qeshta D., Mohamad M., Al Dhaheri A. (2019). Effect of High Fiber Cereal Intake on Satiety and Gastrointestinal Symptoms during Ramadan. Nutrients.

[4] Bardagjy A., Steinberg F. (2019). Relationship Between HDL Functional Characteristics and Cardiovascular Health and Potential Impact of Dietary Patterns: A Narrative Review. Nutrients.

[5] Chooi Y., Ding C., Chan Z., Lo J., Choo J., Ding B., Leow M., Magkos F. (2018). Lipoprotein Subclass Profile after Progressive Energy Deficits Induced by Calorie Restriction or Exercise. Nutrients.

[6] Cheikh Ismail L., Hashim M., Jarrar A.H., Mohamad M.N., Saleh S.T., Jawish N., Bekdache M., Albaghli H., Kdsi D., Aldarweesh D. (2019). Knowledge, Attitude, and Practice on Salt and Assessment of Dietary Salt and Fat Intake among University of Sharjah Students. Nutrients.

[7] Li A., Lee H., Lin Y. (2019). The Effect of Ketoanalogues on Chronic Kidney Disease Deterioration: A Meta-Analysis. Nutrients.

[8] Widomska J., Subczynski W. (2019). Why Is Very High Cholesterol Content Beneficial for the Eye Lens but Negative for Other Organs?. Nutrients.

[9] Yin W., Li Z., Zhang W. (2019). Modulation of Bone and Marrow Niche by Cholesterol. Nutrients.

[10] Idris C., Sundram K., Razis A. (2018). Effect of Consumption Heated Oils with or without Dietary Cholesterol on the Development of Atherosclerosis. Nutrients.

